# Tribute to Dr. Jenner Cruz, an eternal dreamer

**DOI:** 10.1590/2175-8239-JBN-2023-IM002en

**Published:** 2023-11-13

**Authors:** Gianna Mastroianni Kirsztajn, Edison Souza

**Affiliations:** 1 Universidade Federal de São Paulo, Escola Paulista de Medicina, Departamento de Medicina, São Paulo, SP, Brazil.; 2 Universidade do Estado do Rio de Janeiro, Rio de Janeiro, RJ, Brazil.

**Figure 1:**
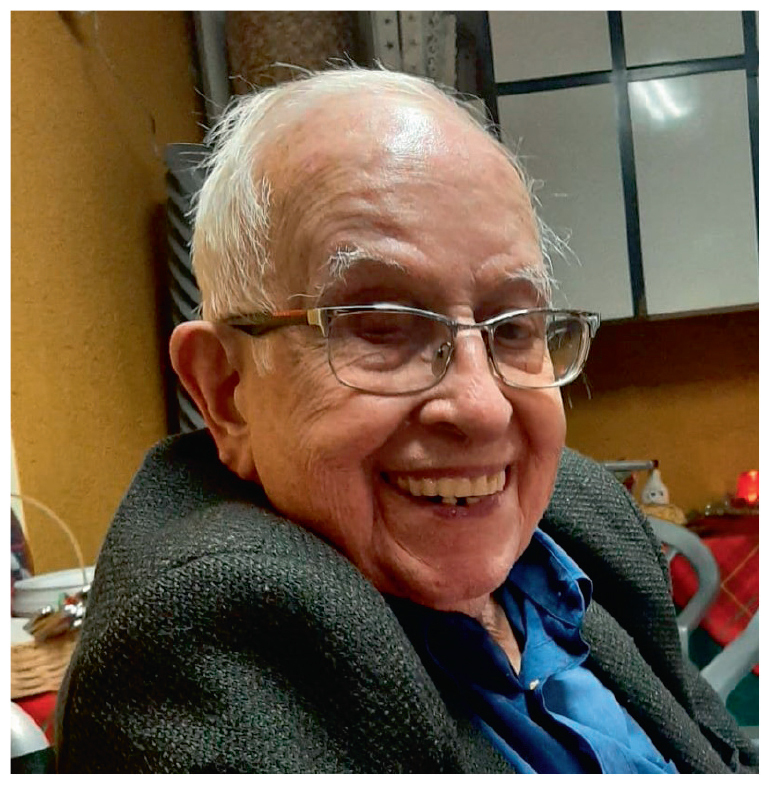
A 2022 photo of Dr. Jenner Cruz, kindly given by his daughter Maria Paula Borges Cruz.

It is not easy to write about someone who touched the lives of many healthcare professionals, patients, friends and family in so many different ways, in which we cannot describe here. But we think that Dr. Jenner Cruz (figure 1) would be disappointed if we didn’t accept the invitation and the opportunity to pay this simple tribute. Notwithstanding, that, after accepting, we (GMK, ES) asked ourselves if we should have done so, since we were not the colleagues who knew him best in his daily life. To try to fill in at least part of the gaps, we contacted colleagues who had spent more time with him than we did, to better portray him.

RAG and SK say that Dr. Jenner was “the son of a medical star from Mogi das Cruzes, Dr. Milton Cruz, and Maria de Lourdes Borges Vieira Cruz. He studied in the city of São Paulo, where he met his wife and lifelong companion, Professor Helga Maria Mazzarolo Cruz. The two studied Medicine at the University of São Paulo (School of Medicine), where they later worked as doctors and professors until mandatory retirement due to old age. He was a full professor at the Nephrology Department of the University of Mogi das Cruzes, SP (1973-2000), and coordinator of the Residency Program in internal medicine (1996-2000). In 1998, he began working at the then newly opened Institute of Nephrology of Mogi das Cruzes, developing care activities in the chronic kidney disease outpatient clinic, until March 2017. He also coordinated weekly clinical meetings at the unit, discussing nephrology clinical cases and topics. He motivated the team and carried out numerous scientific studies”. For them (RAG, SK and team), and certainly for many of us, he “never stopped being a kind, polite and generous Master. He made humility and lightness of spirit his hallmark. He is greatly missed.”

For RBO, “Dr. Jenner Cruz’s work represents, in a special way, the sublime meaning of being a doctor and professor. He practiced medicine for decades and nobly went through all the changes in this area. He always believed in academic life, medical societies and his professional colleagues. He was one of the first doctors in the State to register with CREMESP (CRM no. 159). Collaborated, voluntarily and constructively with the Brazilian Medical Association (AMB), the Association of Medicine of São Paulo (APM), the Society of Nephrology of the State of São Paulo (SONESP) and the Brazilian Society of Nephrology (SBN) throughout his professional career. He has always contributed financially as a member of various national and international medical societies.”

At this point, we remember that he was one of the founding members of SBN and also of SONESP. ES highlights that, on August 2, 1960, together with 115 other colleagues who were interested in the study of kidney diseases and water and electrolyte disorders, he joined the group of SBN founders. JERJ recalls that, in this society, Dr. Jenner held several positions, standing out as treasurer of the National Board and as director and member of the Department of Clinical Nephrology. Certainly, his constant and participative presence in Board meetings and scientific activities was remarkable.

He received many fair and deserved honors during his lifetime (and there is nothing better than being recognized for his actions and works while still alive). We highlight the one who gave its name to the “Jenner Cruz Award”, created by SONESP in 2009, which is awarded at the Congresso Paulista de Nefrologia (State of São Paulo Nephrology Congress) for the best study carried out by residents in the specialty.

It was his idea to create the book Atualidades em Nefrologia^1^ (Updates in Nephrology), which was part of the SBN`s Department of Clinical Nephrology activities, in collaboration with its members. In 1988, the first volume was published and, from then on, a new edition with completely new content was launched every two years at the Brazilian Congress of Nephrology, bringing together chapters from the most diverse authors. Dr. Jenner went to great lengths to promote a wide distribution of invitations for different doctors to participate in these books. Colleagues contributed spontaneously, awaited a selection process and, after acceptance, had their chapters carefully reviewed by Dr. Jenner and collaborators, but above all by him. The Updates in Nephrology book series^1–3^ have become popular due to their recent and/or highly interesting content, and also for giving an opportunity for many young authors.

RBO tells us that Dr. Jenner “encouraged young colleagues to publish the results of their studies in this series, which he believed offered visibility, and therefore value, to the studies of these young people and their mentors. In RBO’s words: “Jenner Cruz was my undergraduate professor. We maintained professional contact for almost three decades. During this period, we edited five books and created the book Emergencies in Nephrology for Clinicians^4^. Above all, I learned, through this interaction, about medicine, courage, humility, determination and family, which he highlighted as being the basis of everything. I never heard him complain about anything, I remember his smile and his kindness. Dr. Jenner Cruz was an exemplary professor, doctor and man.”

Adding some personal impressions here, I (GMK) would like to say that I had the honor of being invited by him to join the group of editors organizing the Updates in Nephrology from the tenth volume^2^ onwards. Volume 16, the last in the series, was published in 2020. It was in this joint production that our friendship really developed, predominantly through written messages. In these books, we all shared some tasks and the review of chapters, respectfully consulting each other in case of doubts. His willingness to create the books was impressive, not only acting in the selection and medical review, but also working personally and in person at the publisher’s headquarters, together with the reviewer, Mrs. Ofélia, on numerous visits to the site. He took on this last task entirely, and each of us had some specific missions, but not so laborious. In total, the Updates in Nephrology series brought together 918 chapters and 1736 authors, including several foreigners (Cameron, Kincaid-Smith, among others), according to a compilation by ES. The colleague also emphasizes that this collection was one of Dr. Jenner’s greatest legacies, possibly the greatest. He highlights that “throughout the volumes we follow the evolution of Nephrology, we see the first publications of many young doctors and we meet famous authors who have already passed away”. ES also reports that he had the privilege of speaking with him four days before his departure, when he was able to express his thanks for the professor’s support and respect.

Everyone who wrote a chapter for the oldest editions of the Updates in Nephrology books received letters from Dr. Jenner. It was incredible! He, himself, wrote almost everything by hand, prepared the envelopes with our names, sent notes and reviews by post and received the responses at his home. Only more recently, after the two youngest co-editors were invited to join the group, these letters stopped being sent.

RBO also comments that “his relative visual limitation resulting from an eye infection in childhood did not prevent him from being an avid reader, which kept him in excellent conditions for scientific updating throughout his career”.

In addition to working as a book organizer/editor, he always wrote his own chapters in these works. GMK considers that he had a peculiar writing style, that is, even when dealing with a scientific issue, he made a point of including whenever possible his professional and/or personal experience, depending on the topic.

His longevity as a doctor is also worth noting, providing care to patients until 2017, as already mentioned. Likewise, even at an advanced age, he continued to be part of postgraduate committees and writing chapters and articles, including papers for the Cultural supplement of the Medical Association of São Paulo (APM).

At this point, we return to talking about Dr. Helga, “a companion on so many journeys”, as mentioned in the presentation of Updates in Nephrology 16, which for GMK was a truly moving text, one of the best. In this edition^3^, Dr. Jenner states that “The Introduction will be dedicated exclusively to her” and, almost at the end, highlights: “I have always had a wonderful life with her”. In another excerpt, she declares: “I, an eternal dreamer, always had a poet to inspire me.” It is clear, in his story and in these words, the importance of Dr. Helga in his trajectory and vice versa.

Finally, to learn about Dr. Jenner’s formal biography, we suggest reading the publication of the São Paulo Academy of Medicine, of which he was a founding member and in which he held the 39th chair since 1979. It contains complete information provided by himself.

We hope to have recalled some of the numerous qualities and contributions of our professor, colleague and nephrologist, Dr. Jenner, whose life and work had a very positive impact on so many, teaching us so much. In this process, we tried to select some aspects of interest, sharing the gratitude and manifestations of affection of different colleagues, as we paid this small tribute to such a special human being.

Names of colleagues (identified by their initials) whose testimonies contributed to this text: Edison Souza (ES); Gianna Mastroianni Kirsztajn (GMK); João Egidio Romão Júnior (JERJ); Rodrigo Bueno Oliveira (RBO); Rui A. Gomes (RAG); Silvana Kesrouani (SK).
